# Study on the Neuroprotective Effects of Eight Iridoid Components Using Cell Metabolomics

**DOI:** 10.3390/molecules29071497

**Published:** 2024-03-27

**Authors:** Bingxian Zhang, Ning Zhou, Zhenkai Zhang, Ruifeng Wang, Long Chen, Xiaoke Zheng, Weisheng Feng

**Affiliations:** 1 College of Pharmacy, Henan University of Chinese Medicine, Zhengzhou 450046, China; 13298186074@163.com (B.Z.); zhoun0813@163.com (N.Z.); zzk15837234321@163.com (Z.Z.); m13938028598@163.com (R.W.); clong0522@163.com (L.C.); 2 The Engineering and Technology Center for Chinese Medicine Development of Henan Province, Zhengzhou 450046, China; 3 Collaborative Innovation Center of Research and Development on the Whole Industry Chain of Yu-Yao, Zhengzhou 450046, China; 4 Collaborative Innovation Center for Chinese Medicine and Respiratory Diseases Co-Constructed by Henan Province & Education Ministry of P.R. China, Zhengzhou 450046, China

**Keywords:** iridoid, PC12 cell, neuronal injury, cell metabolomics, UPLC-Q/TOF-MS

## Abstract

Iridoid components have been reported to have significant neuroprotective effects. However, it is not yet clear whether the efficacy and mechanisms of iridoid components with similar structures are also similar. This study aimed to compare the neuroprotective effects and mechanisms of eight iridoid components (catalpol (CAT), genipin (GE), geniposide (GEN), geniposidic acid (GPA), aucubin (AU), ajugol (AJU), rehmannioside C (RC), and rehmannioside D (RD)) based on corticosterone (CORT)-induced injury in PC12 cells. PC12 cells were randomly divided into a normal control group (NC), model group (M), positive drug group (FLX), and eight iridoid administration groups. Firstly, PC12 cells were induced with CORT to simulate neuronal injury. Then, the MTT method and flow cytometry were applied to evaluate the protective effects of eight iridoid components on PC12 cell damage. Thirdly, a cell metabolomics study based on ultra-performance liquid chromatography–quadrupole–time-of-flight mass spectrometry (UPLC-Q/TOF-MS) was performed to explore changes in relevant biomarkers and metabolic pathways following the intervention of administration. The MTT assay and flow cytometry analysis showed that the eight iridoid components can improve cell viability, inhibit cell apoptosis, reduce intracellular ROS levels, and elevate MMP levels. In the PCA score plots, the sample points of the treatment groups showed a trend towards approaching the NC group. Among them, AU, AJU, and RC had a weaker effect. There were 38 metabolites (19 metabolites each in positive and negative ion modes, respectively) identified as potential biomarkers during the experiment, among which 23 metabolites were common biomarkers of the eight iridoid groups. Pathway enrichment analysis revealed that the eight iridoid components regulated the metabolism mainly in relation to D-glutamine and D-glutamate metabolism, arginine biosynthesis, the TCA cycle, purine metabolism, and glutathione metabolism. In conclusion, the eight iridoid components could reverse an imbalanced metabolic state by regulating amino acid neurotransmitters, interfering with amino acid metabolism and energy metabolism, and harmonizing the level of oxidized substances to exhibit neuroprotective effects.

## 1. Introduction

PC12 cells, a pheochromocytoma cell line derived from rat adrenal medulla, have classical neuronal properties including high levels of neurotransmitter secretion, characteristics of neuronal morphology, and the expression of ion and neurotransmitter receptors [1]. At present, PC12 cells are widely used in neurobiology research, and different modeling drugs can simulate various disease states [2]. Glucocorticoids (GCs) can regulate many kinds of physiological processes, including energy metabolism, inflammation, and stress responses, but excessively elevated GCs will cause detrimental effects on CNS function. Corticosterone (CORT) acts as a major GC; its sustained exposure can decrease serotonin (5-hydroxytryptamine, 5-HT) release and lead to pathological hippocampal damage [3,4,5]. Therefore, high-concentration CORT-induced models are usually used as in vitro experimental models to mimic neuronal injury in depression [6]. Fluoxetine (FLX), a selective 5-HT reuptake inhibitor (SSRI) and typical antidepressant, has been shown to provide neuroprotective effects in central nervous system injury [7,8,9]. Therefore, fluoxetine was used as the positive control in this study.

In recent years, Chinese herbal medicine has received a great deal of attention for its neuroprotective effects [10,11]. Iridoids, a major category of monoterpenoids isolated from various traditional medicinal plants, especially prevalent in the family Scrophulariaceae [12,13], have been proven to have significant neuroprotective effects [14], including in relation to depression [15,16], Alzheimer’s disease (AD) and Parkinson’s disease (PD) [17], anxiety [18], ischemic stroke [19], etc. Previously, our team has carried out extensive studies on the neuroprotective effects of *Rehmannia glutinosa* Libosch (RG) and its active components. It has been revealed that echinacoside could improve hippocampal neurogenesis via the CREB-BDNF signaling pathway [20]; aucubin could inhibit LPS-induced neuroinflammation by modulating the release of inflammatory factors [21]; rehmangoside D could protect PC-12 cells from corticosterone-induced injury by activating the BDNF-Trk B pathway and inhibiting apoptosis pathways [22]. However, whether the efficacy and mechanisms of iridoid components with similar structures are also similar is not clear.

Cell metabolomics is an emerging field that is widely recognized as a reflection of cellular metabolic phenomena [23]. In this field, cells are taken as the object of study and intracellular metabolites and pathways are analyzed. This is to help distinguish normal and abnormal cellular states, and thus reveal the metabolic changes of cells after being exposed to external stimuli [24,25]. Cells are the basic units that make up the structure and function of an organism [26]. An in vitro cell model of a single pathological state facilitates the interpretation of experimental results and the exploration of the action mechanism [27]. Therefore, cell metabolomics could reflect the differences in efficacy and mechanisms by comparing the administration groups in parallel.

**Figure 1:**
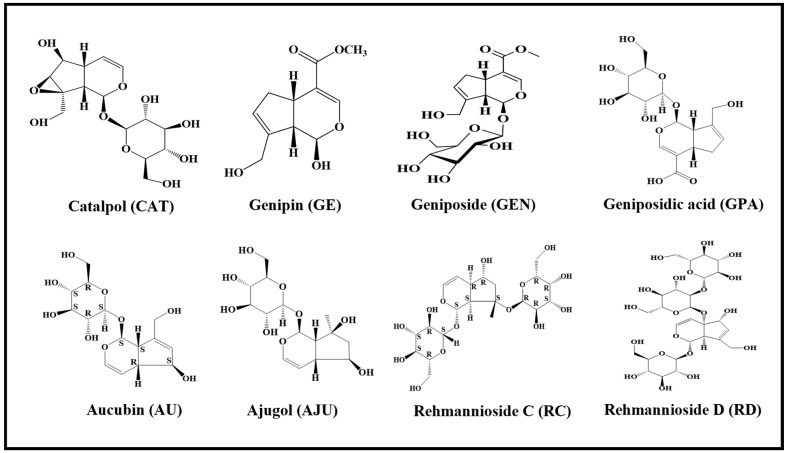
Structures of eight active iridoid components.

Based on previous research work, firstly, a model of CORT-induced PC12 cells was established to simulate neuronal damage. Secondly, based on the preliminary separation basis of Chinese medicinal plants in our team, and the iridoid components found to have great biological activity in a literature review, eleven iridoid components were selected. The MTT method and flow cytometry were performed to assess the neuroprotective effects of iridoid components. As a result, eight active iridoid components were selected for subsequent cellular experiments (the structures of the eight iridoid components are shown in Figure 1). Thirdly, a cell metabolomics approach was employed to screen out potential biomarkers of neuroprotection and construct the relevant pathway network. Finally, the neuroprotective effects and mechanisms of eight iridoid components on PC12 cells were analyzed and compared, providing a basis for the development of new drugs.

## 2. Results

### 2.1. The Results of MTT Assay and Flow Cytometry

**Figure 2:**
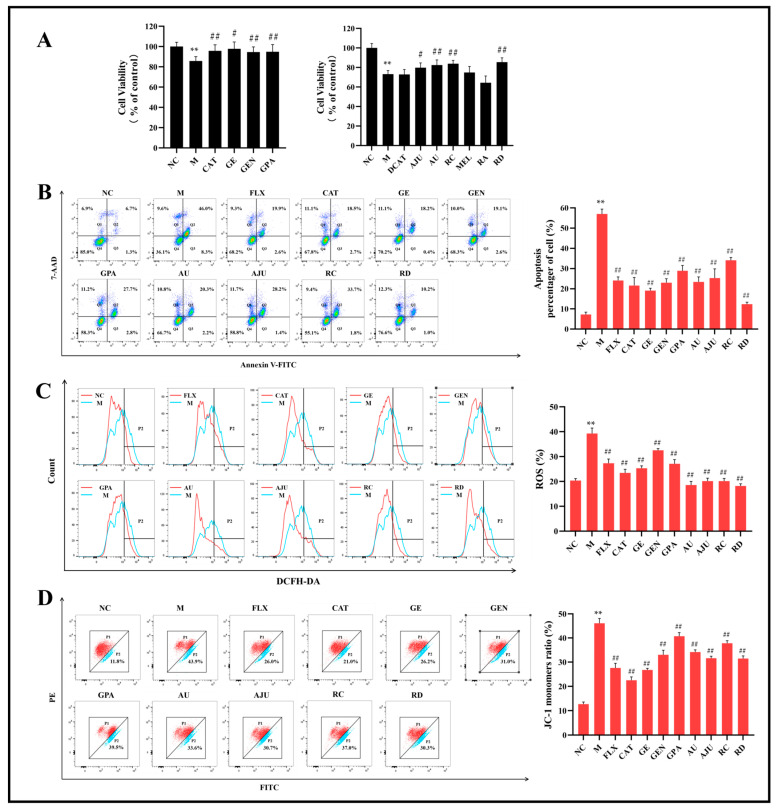
Interventional effects of eight iridoid components on CORT-induced PC12 cells. (**A**) Effect of eleven iridoid components on cell viability in CORT-induced PC12 cells (mean ± SD, *n* = 6 per group). (**B**–**D**) Effects of eight iridoid components on cell apoptosis, intracellular ROS levels, and MMP levels in CORT-induced PC12 cells (mean ± SD, *n* = 3 per group). In (**B**), pseudocolour shows the density of cells by shades of colour. In (**D**), blue fluorescence represents JC-1 monomer and red fluorescence represents JC-1 in polymer form. ** p < 0.01, compared with NC group; *^#^ p* < 0.05, *^##^ p* < 0.01, compared with M group.

As shown Figure 2A, the results of the MTT assay showed that the viability of PC12 cells in the M group was significantly reduced (*p* < 0.01) compared with the NC group, and the eight iridoid component groups (CAT/GE/GEN/GPA/AU/AJU/RC/RD) of the eleven treatment groups improved the viability of PC12 cells to different degrees (*p* < 0.05 or *p* < 0.01). Then, the cell apoptosis rate, ROS, and MMP levels in PC12 cells were determined by flow cytometry to further verify the neuroprotective effects of the eight iridoid components.

As shown Figure 2B–D, the results of the flow cytometry showed that the eight iridoid components (CAT/GE/GEN/GPA/AU/AJU/RC/RD) effectively inhibited the level of cell apoptosis and increased cell viability (*p* < 0.01), reduced intracellular ROS levels and alleviated oxidative stress damage (*p* < 0.01), significantly reduced the level of JC-1 monomers, and elevated the level of MMP (*p* < 0.01) in PC12 cells induced with CORT. Therefore, in the subsequent experiment, the eight active components were used to carry out a cell metabolomics study.

### 2.2. Method Validation Results

As shown in Figure S1, the base peak chromatograms (BPCs) were obtained with the optimized UHPLC-MS system method in positive- and negative-source ion modes, which exhibited a high degree of separation between the chromatographic peaks. As shown in Table S1, the RSDs of the retention times and peak areas of 38 metabolites in the QC samples were less than 5%, proving that the stability and repeatability of the system were reliable.

### 2.3. Metabolic Profiles of PC12 Cell Samples

**Figure 3:**
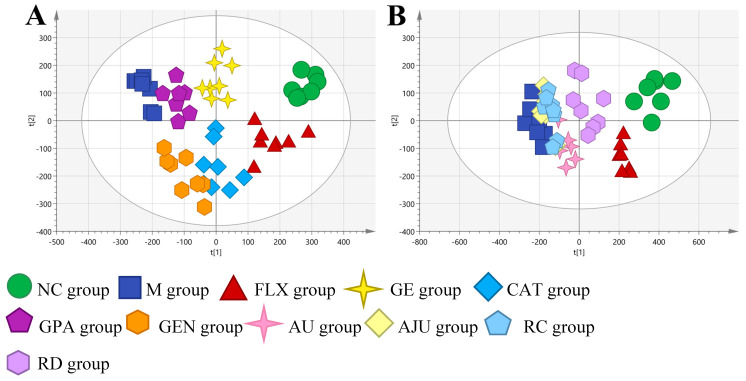
The PCA score plots obtained from NC, M, and eight treatment groups. (**A**) NC, M, FLX, GE, CAT, GPA and GEN groups in the first stage. (**B**) NC, M, FLX, AU, AJU, RC and RD groups in the second stage.

The PCA method was performed to describe the metabolic profiles of groups including the normal control group (NC), CORT-induced group (M), positive drug group (FLX), and the eight iridoid administration groups (CAT/GE/GEN/GPA/AU/AJU/RC/RD). Due to the excessive number of experimental samples, the study was conducted in two stages. The experimental subjects in the first stage were the NC, M, FLX, CAT, GE, GEN, and GPA groups. As shown in Figure 3A and Figure S2A–E, the sample points of each group were, respectively, clustered into one category. There was a significant separation between the NC and M group, which suggested that the metabolic profile of the NC group changed after CORT intervention. In addition, the metabolic profile of the FLX group had a tendency towards the NC group and was far from the M group, suggesting that the neuronal injury model of PC12 cells was successfully established. As for the treatment groups, the sample points of the CAT, GE, GEN, and GPA groups were between the NC and M group. In the second stage, AU, AJU, RC, and RD could also regulate the metabolic disorders of PC12 cells to various degrees, as shown in Figure 3B and Figure S2F–I. Therefore, the eight components could ameliorate PC-12 cell injuries induced by CORT. However, the RD group was closer to the NC group than the AU, AJU, and RC groups. The three groups were slightly removed from the metabolic profile of the M group, which indicates that AU, AJU, and RC have a weaker effect on regulating the model.

### 2.4. Identification of Potential Biomarkers

**Figure 4:**
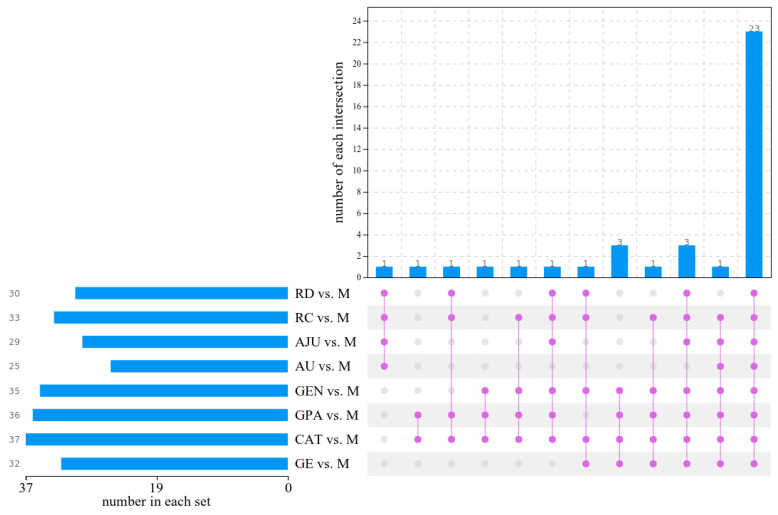
UpSet plots of differential biomarkers in the eight iridoid groups. The horizontal bar graph on the left side indicates the number of markers in each group, the connecting lines among points in the middle array indicate specific intersections in different groups, and the vertical bar graph indicates the number of markers corresponding to intersections.

As shown in Figure S3, based on the PCA score plots, an OPLS-DA model was established to search for endogenous metabolites causing differences between the NC group and the M group, as well as the M group and the eight iridoids administration groups. In the positive and negative ion modes, there was an obvious separation between the M group and the N/CAT/GE/GEN/GPA/AU/AJU/RC/RD groups. Two hundred permutation tests indicated that all R^2^ and Q^2^ values were lower than the original point, and the intercept of the regression line of Q^2^ in the vertical coordinate was a negative value. Thus, the OPLS-DA model was well fitted and reliable. In the S-plots, the red points (VIP > 3, *p* < 0.05) further from the origin contributed more to the difference between two groups. Combining the HMDB and KEGG databases, there were 32/37/36/35/25/29/33/30 differential metabolites in the GE/CAT/GPA/GEN/AU/AJU/RC/RD groups, respectively (Figure 4). After removing duplicate metabolites, a total of 38 differential metabolites (19 metabolites each in the positive and negative ion modes, respectively) were found, as listed in Table S2, among which 23 metabolites (12 and 11 metabolites in the positive and negative ion modes, respectively) were common biomarkers of the eight iridoids groups, suggesting that the metabolites by which they regulated the cellular injury model were similar. Meanwhile, the EICs and MS spectra of pooled QCs for metabolites that co-eluted within the first minute in positive and negative ion modes, respectively, are shown in Figure S4.

**Figure 5:**
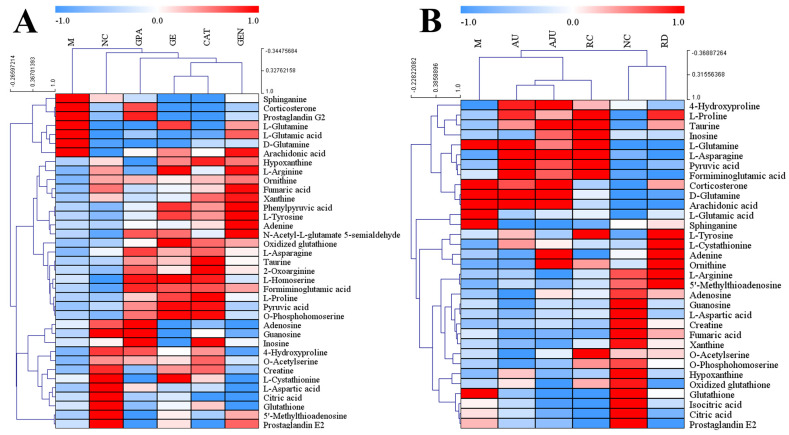
Cluster analysis of potential biomarkers in the groups (Pearson correlation was used for clustering, average linkage clustering was used for the clustering method, and double gradient was used for gradient style). (**A**) NC, M, FLX, GE, CAT, GPA and GEN groups in the first stage. (**B**) NC, M, FLX, AU, AJU, RC and RD groups in the second stage. Each row represents a metabolite, and each column represents an experimental group (N/M/CAT/GE/GEN/GPA/AU/AJU/RC/RD). The color from red to blue represents the average content from high to low.

To learn the specific changes in the metabolites, a relative quantification of biomarkers was carried out using QuantAnalysis 4.4 software, and a hierarchical clustering heatmap was used to visualize the results of the average content of metabolites in each group. As shown in Figure 5, compared with the NC group, the content of metabolites showed significant increases or decreases in the M group, while there was a trend of regression toward the NC group in all eight treatment groups. In Figure 5A, the CAT, GE, GEN, GPA, and NC groups are first clustered into one category, and then the M group and the five groups are clustered. The results suggest that the four iridoid components have a good effect against the CORT-induced M group. In Figure 5B, the RD and NC groups are clustered into one category, whereas AU, AJU, RC, and M are clustered into another category. The result suggests that the RD group might have a stronger effect against the M group than AU, AJU, or RC. The above clustering results are generally consistent with the trend of separation in the PCA score plot (Figure 3).

### 2.5. Metabolic Pathway Analysis

**Figure 6:**
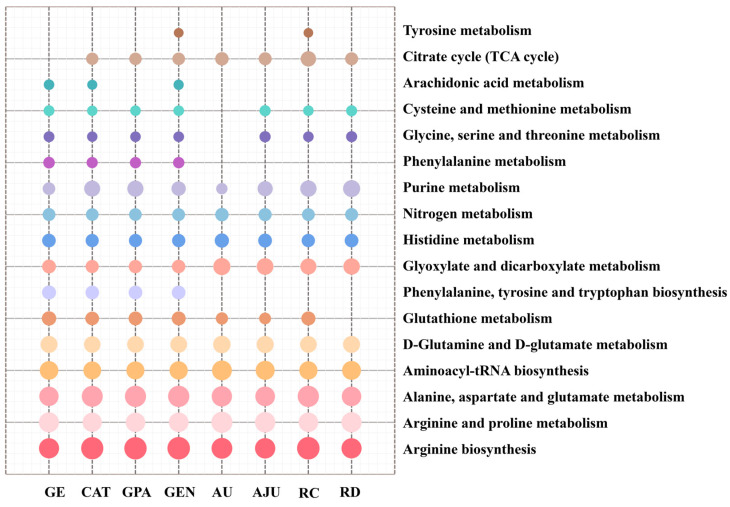
Metabolic pathway enrichment in GE/CAT/GPA/GEN/AU/AJU/RC/RD groups. (*p* < 0.05; the size of dots represents the impact factor of the metabolic pathway).

**Figure 7:**
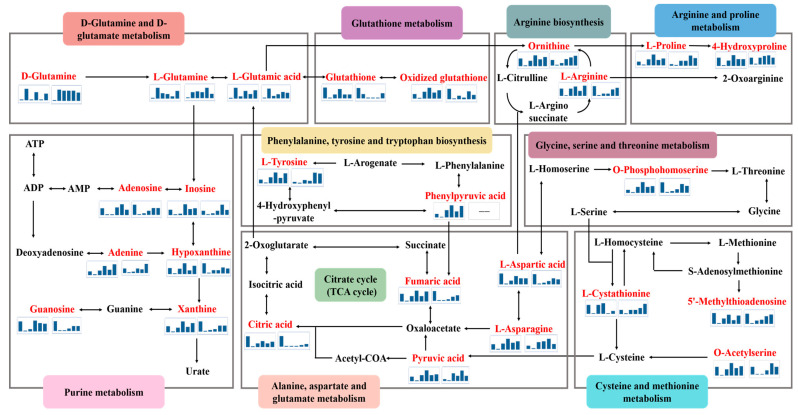
Metabolic network associated with neuroprotective effects of iridoids. Mini bar chart from left to right represents the average content of biomarkers in NC, M, GE, CAT, GPA, and GEN groups in the first stage and NC, M, AU, AJU, RC, and RD groups in the second stage. Metabolites labeled in red were from cell samples.

To further explore the potential neuroprotective mechanisms of GE/CAT/GPA/GEN/AU/AJU/RC/RD, the identified metabolites in each treatment group were, respectively, enriched and analyzed with MetaboAnalyst 5.0 and MBRole 2.0 data-processing platforms. As shown in Figure 6, there were seventeen involved metabolic pathways with a *p* < 0.05, nine of which were co-intervened by the eight treatment groups, including alanine, aspartate and glutamate metabolism, arginine and proline metabolism, D-glutamine and D-glutamate metabolism, purine metabolism, and glutathione metabolism. More than half of the pathways suggested that there was a high degree of similarity in the metabolic pathways interfered with by the eight iridoid components. According to the KEGG database, the metabolic network related to the neuroprotective effects of eight iridoid components was mapped, as shown in Figure 7. Upon analysis, it was found that the perturbed pathways were mainly related to energy metabolism, oxidative metabolism, and amino acid metabolism. Meanwhile, the network visually revealed the conversion relationships between critical biomarkers.

## 3. Discussion

In previous work, our team has reported the neuroprotective effects of RG and its active components [20,21,22]. However, whether the efficacy and mechanisms of iridoid components with similar structures are also similar is not clear. Therefore, PC12 cells induced by CORT were used to conduct the experiments. In the results of the MTT assay and the flow cytometry, it was found that CAT/GE/GEN/GPA/AU/AJU/RC/RD can improve the viability of PC12 cells, inhibit the level of cell apoptosis, reduce intracellular ROS levels, and increase the level of MMP. In order to further explore and compare the mechanism of action, cell metabolomics was carried out. The result suggested that the eight iridoid components mainly regulated the D-glutamine and D-glutamate metabolism, arginine biosynthesis, TCA cycle, purine metabolism, and glutathione metabolism to exert neuroprotective effects.

### 3.1. D-Glutamine and D-Glutamate Metabolism

Glutamate is the major excitatory neurotransmitter in the central nervous system and has high concentrations in brain tissue [28]. Glutamate plays a vital role in the growth and development of the brain by facilitating the transport of Ca^2+^ [29,30]. It is known that the accumulation of excessive glutamate causes the overstimulation of glutamate receptors and a large amount of Ca^2+^ to inwardly flow into the intracellular space, leading to excitatory neurotoxicity [31,32]. In addition, glutamate and glutamine are interconverted to form the glutamate–glutamine neurotransmitter cycle in the mitochondria of brain cells. Therefore, the metabolic regulation of the two metabolites is critical to the maintenance of dynamic balance in the cells [33]. In the present study, the abnormal elevation of glutamate and glutamine levels may have provoked neuronal cell death and induced D-glutamine and D-glutamate metabolic disorders in the M group [34]. After the administration of CAT, GE, GEN, GPA and RD, the situation was reversed and the balance between intracellular and extracellular glutamate/glutamine was restored. In the AU, AJU, and RC groups, the levels of L-glutamate were significantly reversed, but there was no obvious decrease in L-glutamine levels, and a weak retracement of D-glutamine. Thus, the roles of AU, AJU, and RC in regulating the pathway may be less powerful. Put simply, the eight iridoids could lower glutamate concentration and inhibit Ca^2+^ influx, thereby effectively protecting PC12 cells from CORT-induced neuronal injury and metabolic disorders.

### 3.2. Arginine Biosynthesis

Arginine metabolism contains two main branches: one branch is involved in the urea cycle to produce ornithine and urea, and the other one is involved in the nitric oxide (NO) cycle to produce citrulline and NO [35,36]. NO is an important neurotransmitter that has been associated with the pathophysiology of various neurological disorders. Increased levels of NO have been reported in depressive disorder [37,38]. In our study, decreased levels of arginine and ornithine were observed in the M group. Thus, we speculated that arginine may be more involved in the NO cycle to generate NO, denoting neuronal injury. After the administration of CAT, GE, GEN, GPA, AU, AJU, RC, and RD, arginine levels were reversed to different extents. In particular, the arginine levels were clearly less increased in the AU and AJU groups than in the other administration groups. Therefore, arginine biosynthesis is of great significance for maintaining NO balance, and the eight iridoids could exert a similar neuroprotective mechanism by regulating amino acid neurotransmitters.

### 3.3. Citric Acid Cycle (TCA Cycle)

The tricarboxylic acid cycle (TCA), also called the citric acid cycle, is known as a central metabolic hub which can maintain cells in a steady state [39]. The TCA cycle begins with the condensation of acetyl-COA with oxaloacetate to form citric acid. This cycling process produces ATP, which provides energy for the cells [40,41]. Thus, citric acid, as the first intermediate in the TCA cycle, performs a critical role in the energy metabolism [42]. Moreover, changes in pyruvate levels, another important metabolite of the TCA cycle and also a key substrate for the three major energy metabolisms (carbohydrates, fats, and amino acids), affect cellular energy supply [43]. Furthermore, there is a close association between neuronal activity and energy metabolism [44]. In the present study, the levels of pyruvic acid, citric acid, and fumaric acid in the M group were decreased compared to the NC group. That is to say, the blocked TCA cycle affected the energy supply to cells and neuronal damage may have been induced. After the administration of CAT, GE, GEN, GPA, AU, AJU, RC, and RD, there was an upward trend in metabolite levels in the treatment groups. The elevation of citric acid and fumaric acid in the AU, AJU, and RC groups were not significant compared to the M group, but all of the eight components had an apparent pullback effect on the pyruvate levels. Therefore, the eight iridoid components could improve energy metabolic disorders and exert neuroprotective effects by regulating the TCA cycle.

### 3.4. Purine Metabolism

Purine mainly exists in the form of nucleotides and participates in various cellular processes to maintain metabolic homeostasis [45]. In purine metabolism, adenosine is deaminated under the action of adenosine deaminase to produce inosine, which is further converted to obtain hypoxanthine, which is then catalyzed by xanthine oxidase to produce xanthine, and the final metabolite uric acid [46]. Thus, the process by which xanthine oxidase catalyzes hypoxanthine and xanthine could produce reactive oxygen species (ROS) and hydrogen peroxide (H_2_O_2_), resulting in increased oxidative stress [47,48]. In this study, decreased levels of adenosine, inosine, hypoxanthine, and xanthine were observed in the M group. It was speculated that the overactivity of xanthine oxidase would lead to the production of excess oxidative substances, resulting in increased oxidative damage and neuronal apoptosis [49]. After the administration of CAT, GE, GEN, GPA, AU, AJU, RC, and RD, purine metabolite levels increased to different degrees, suggesting that the eight iridoid components play an important role in improving neuronal injury by restoring the dysregulation of purine metabolism and reducing the production of oxidative substances. Of these, GE and AU showed a lesser degree of improvement in terms of levels of purine metabolites.

### 3.5. Glutathione Metabolism

Glutathione (GSH) not only acts as a critical antioxidant in cells, but also plays the function of neurotransmission and modulation in the central nervous system. It has been reported that reduced intracellular GSH viability may disrupt redox homeostasis and promote ROS accumulation, thereby inducing oxidative stress in cells, ultimately resulting in neuronal damage [50,51,52]. In the present study, the GSH level in the M group was significantly decreased. After drug intervention, the GSH level in the CAT, GE, GEN, GPA, and RD groups increased, and the other three treatment groups (including the AU, AJU, and RC groups) did not show a significant upward trend. However, glutathione metabolism is only one of the pathways that AU, AJU, and RC protect against cellular damage, and the impact factor of glutathione metabolism is not the greatest. Moreover, AU, AJU, and RC can also exert antioxidant effects by regulating purine metabolism, as mentioned above. Therefore, the eight iridoid components exhibit similar neuroprotective mechanisms.

In summary, the neuroprotective effects and mechanisms of CAT/GE/GEN/GPA/AU/AJU/RC/RD were compared in this study. The results of the activity screening suggested that the eight iridoid components could improve the survival of PC12 cells. The cell metabolomics research indicated that the eight iridoid components exert neuroprotective effects mainly by regulating amino acid neurotransmitters, interfering with amino acid metabolism and energy metabolism, and harmonizing the level of oxidized substances. This study not only demonstrates the neuroprotective effects of iridoid components on CORT-induced neuronal injury, but also provides a basis for the development of new drugs.

## 4. Materials and Methods

### 4.1. Chemicals and Reagents

Dihydrocatalpol (DCAT), melittoside (MEL), genipin (GE), geniposide (GEN), geniposidic acid (GPA), rehmannioside A (RA), and rehmannioside D (RD) were purchased from Shanghai Yuanye Bio-Technology Co., Ltd. (Shanghai, China). Catalpol (CAT), aucubin (AU), ajugol (AJU), and rehmannioside C (RC) were purchased from Sichuan Victory Biological Technology Co., Ltd. (Sichuan, China). Corticosterone (CORT) was purchased from Cayman Chemical company (Ann Arbor, MI, USA). Fluoxetine (FLX) was a positive drug purchased from MedChemExpress (Monmouth Junction, NJ, USA). RPMI medium 1640 was obtained from Gibco (Grand Island, NY, USA). Fetal bovine serum (FBS) was obtained from Zhejiang Tianhang Bio-Technology Co., Ltd. (Zhejiang, China). HPLC-grade acetonitrile and methanol were supplied by Fisher Chemical (Waltham, MA, USA). Formic acid was supplied by Fisher Scientific (Waltham, MA, USA). Ultrapure water was purified with Arium^®^ Pro Ultrapure Water Systems (Sartorius Stedim Biotech, Göttingen, Germany).

### 4.2. Cell Culture and Treatment

Rat pheochromocytoma PC12 cells were obtained from the Cell Bank of Chinese Academy of Sciences (Shanghai, China). PC12 cells were cultured in RPMI-1640 with 10% FBS containing 1% penicillin–streptomycin–amphotericin B solution. The cells were grown at 37 °C in a humidified incubator at 5% CO_2_. PC12 cells were seeded in culture plates/dishes and cultured for 24 h. Then, the cells were divided into 14 groups as follows: the normal control group (NC) was incubated with medium; the CORT group (M) was induced with 500 µM CORT only; the positive drug group (FLX) was exposed to medium containing 0.01 µM fluoxetine and 500 µM CORT; 11 iridoid administration groups (CAT, DCAT, GE, GEN, GPA, MEL, AU, AJU, RA, RC, RD) were treated with medium containing 10 µM catalpol, dihydrocatalpol, genipin, geniposide, geniposidic acid, melittoside, aucubin, ajugol, rehmannioside A, rehmannioside C, rehmannioside D, and 500 µM CORT. The cells of each group were incubated at 37 °C in 5% CO_2_ for another 24 h.

### 4.3. Cell Viability Assay with MTT

PC12 cells were seeded in 96-well plates at a concentration of 4 × 10^4^ cells/mL (*n* = 6 parallel wells per group). After the complete adherence of cells, the medium was removed and the cells were re-cultured for 24 h with different treatment groups (NC, M, CAT, DCAT, GE, GEN, GPA, MEL, AU, AJU, RA, RC, RD, 10 µM). Then, 20 µL of 5 mg/mL MTT solution was added into each well for 4 h in the incubator. After that, the medium was discarded and 150 µL DMSO was added to each well. Finally, the absorbance was measured using a microplate reader at 490 nm. The obtained data were analyzed statistically with SPSS 24.0 software and Graphpad Prism 8.0 software to express cell viability.

### 4.4. Flow Cytometry Analysis of Intracellular Cell Apoptosis, ROS, and MMP

PC12 cells were seeded at a concentration of 8×10^4^ cells/well in 6-well plates (*n* = 3 parallel wells per group) for 24 h. The cells were randomly divided into the NC, M, and FLX groups and the eight iridoid groups (CAT/GE/GEN/GPA/AU/AJU/RC/RD, 10 µM) obtained by MTT for activity. After 24 h of drug treatment, the cells were trypsinized and collected by centrifugation. The proportion of apoptotic cells was analyzed by flow cytometry according to the Annexin V-FITC/7-AAD apoptosis kit (Elabscience, Wuhan, China) manual. The ROS levels were determined by flow cytometry according to the reactive oxygen species assay kit (Solarbio, Beijing, China) manual. The evaluation of MMP was determined by flow cytometry according to the Mitochondrial Membrane Potential assay kit with the JC-1 (Beyotime, Shanghai, China) manual.

### 4.5. Cell Collection and Sample Preparation for LC-MS

PC12 cells in the exponential growth phase were inoculated in 100 mm culture dishes and were randomly divided into the NC group, M group, FLX (positive drug) group, and eight treatment groups (CAT, GE, GEN, GPA, AU, AJU, RC, and RD) (*n* = 8). Cells were cultured and administered as described above. After treatment, the medium was removed and cells were washed three times with pre-cooled PBS. To suppress cell metabolism, the cells were immediately quenched with liquid nitrogen. As the optimized extraction solvent, 1 mL ice-cold acetonitrile/methanol/water mixed solution (2:2:1, *v*/*v*/*v*) was added to culture dishes; the cells were collected with a cell scraper to obtain cell suspension and were transferred to 1.5 mL Eppendorf tubes. Next, each sample was vortexed for 60 s and lysed over three repeated freeze–thaw cycles in liquid nitrogen at 37 °C (freezing and thawing for 3 min, respectively) to obtain as many intracellular metabolites as possible. After storage in a −20 °C refrigerator for 1 h to precipitate the protein, the samples were centrifuged at 13,000 rpm for 15 min at 4 °C. Then, the supernatants were collected and dried with a speed vacuum concentrator (SAVANT SPD2010, Thermo Fisher Scientific, Waltham, MA, USA) to obtain the residues, which were re-dissolved with 80 µL acetonitrile/water (1:1, *v*/*v*). The samples were processed in an ultrasonic bath at 4 °C for 20 min and centrifuged at 13,000 rpm for 15 min at 4 °C. Finally, 50 µL supernatants were removed into injection vials for LC-MS analysis. In addition, 5 µL of each sample was mixed as a quality control (QC) sample to test the stability and repeatability of the whole system.

### 4.6. UHPLC-Q/TOF-MS Analysis

Metabolomics analysis was carried out on a Dionex UltiMate 3000 system (Thermo Scientific, USA) combined with a maXis HD Q-TOF mass spectrometer equipped with an electrospray ionization (ESI) source (Bruker, Germany). The separation of cell samples was based on an Acclaim™ RSLC 120 C18 chromatographic column (2.1 mm × 100 mm, 2.2 μm) and the column temperature was maintained at 40 °C. The 2 µL injection volume of each sample was taken with an automatic sampler. The mobile phase consisted of ultrasonic acetonitrile (A) and water containing 0.1% formic acid (B) and the flow rate was set at 0.3 mL/min. The optimized conditions of gradient elution were as follows: 0–5 min, 5–60% A; 5–20 min, 60–95% A; 20–22 min, 95–5% A. An electrospray ionization source was used to detect the samples in the positive and negative ion modes, respectively. The optimized performance parameters were as follows: Na formate flow rate, 45 µL/h; mass scan range, 50–1500; pressure of nebulizer gas, 2.0 Bar; dry gas temperature, 230 °C; dry gas flow rate, 8 L/min; capillary voltage, 3500 V (ESI^+^) and 3 200 V (ESI^−^). After UHPLC-Q/TOF-MS analysis, the base peak chromatogram (BPC) was obtained as the strongest ion intensity at each time point. Then, the chromatogram of certain mass-to-charge ratio (*m*/*z*) was extracted based on the BPCs of the QC samples to obtain the extracted ion chromatogram (EIC).

### 4.7. Method Validation

In order to reach the best separation effect of the cell samples and improve the sensitivity of instrument detection, the conditions of the column, mobile phase, and elution gradient were optimized. The base peak chromatograms (BPCs) were obtained with the optimized UHPLC-MS system method in positive- and negative-source ion modes. To evaluate the stability of the system, 38 metabolites in the positive and negative ion modes were selected from the QC samples, and the relative standard deviations (RSDs) of the retention times and peak areas were calculated.

### 4.8. Data Processing and Analysis

Raw data collected by UHPLC-Q/TOF-MS were pre-processed with Profile Analysis 2.1 (Bruker, Germany), including peak correction, peak alignment, noise reduction, data normalization, etc. The obtained Bucket table, containing retention time and *m*/*z* information in positive and negative ion modes, was merged and imported into SIMCA-P 14.1 software (Umetrics AB, Vasterbottens Lan, Sweden) for dimension reduction and multivariate statistical analysis, including unsupervised principal component analysis (PCA) and supervised orthogonal partial least squares discriminant analysis (OPLS-DA). PCA was used to analyze the metabolic profile of each group, and an OPLS-DA model was established to distinguish the differences between groups. Furthermore, permutation tests were performed 200 times to validate the reliability of the OPLS-DA models. Based on the OPLS-DA model, the contribution of each metabolite to the differences between groups was evaluated with Variable Importance in the Project (VIP). Potential biomarkers were preliminarily screened by *t*-test (*p* < 0.05), VIP value (VIP > 3) and Fold Change (FC ≥ 1.2 or FC ≤ 0.83). Then, further identification was performed by comparing the retention time and mass-to-charge ratio (*m*/*z*) information with online databases such as the Human Metabolome Database (https://hmdb.ca/, accessed on 14 February 2023), MassBank (https://massbank.eu/MassBank/, accessed on 14 February 2023), and METLIN (https://metlin.scripps.edu/, accessed on 14 February 2023). Finally, the biomarkers were correctly annotated in combination with MS/MS fragments and relevant published literatures. In addition, the semi-quantitative results of each metabolite were imported into MeV 4.8.0 software to draw the heat map. Finally, MetaboAnalysis (https://www.metaboanalyst.ca, accessed on 10 April 2023) and KEGG (https://www.genome.jp/kegg/, accessed on 10 April 2023) databases were used for the enrichment analysis of metabolic pathways and the construction of the metabolic network.

## 5. Conclusions

CAT, GE, GEN, GPA, AU, AJU, RC, and RD could exert neuroprotective effects. Although there were differences in the extent to which the eight iridoid components improved metabolites levels, and particularly AU, AJU, and RC had weakly regulated effects overall, the eight iridoid components have basically similar mechanisms of action, including regulating amino acid neurotransmitters, interfering with amino acid metabolism and energy metabolism, and harmonizing levels of oxidized substances.

## Data Availability

Data are contained within the article or Supplementary Materials.

## Supplementary Materials

The following supporting information can be downloaded at: https://www.mdpi.com/article/10.3390/molecules29071497/s1, Figure S1: BPC profile of QC cell sample in positive (A) and negative (B) electrospray ionization mode. Figure S2: PCA score plots obtained from NC, M, and eight treatment groups (NC, GE, CAT, GPA, GEN, AU, AJU, RC, RD, VSM). Figure S3: OPLS-DA scores, S-plots, validation plots derived from NC, M, and eight treatment groups (NC, GE, CAT, GPA, GEN, AU, AJU, RC, RD, VSM). Figure S4: The EICs and MS spectra of pooled QCs for metabolites that co-eluted within the first minute in positive and negative ion modes, respectively. Table S1: RSDs of retention time and peak area of 38 metabolites in QC samples. Table S2: Summary of differential biomarkers. Table S3: Fold Change report of differential biomarkers.
