# Supplementary material for: Study on the Neuroprotective Effects of Eight Iridoid Components Using Cell Metabolomics
Source: Molecules. 2024 Mar 27;29(7):1497. doi: 10.3390/molecules29071497 (PMC11013420; doi:10.3390/molecules29071497)
Supplement: Supplementary file 1 [file molecules-29-01497-s001.zip › molecules-2836492-supplementary.pdf]

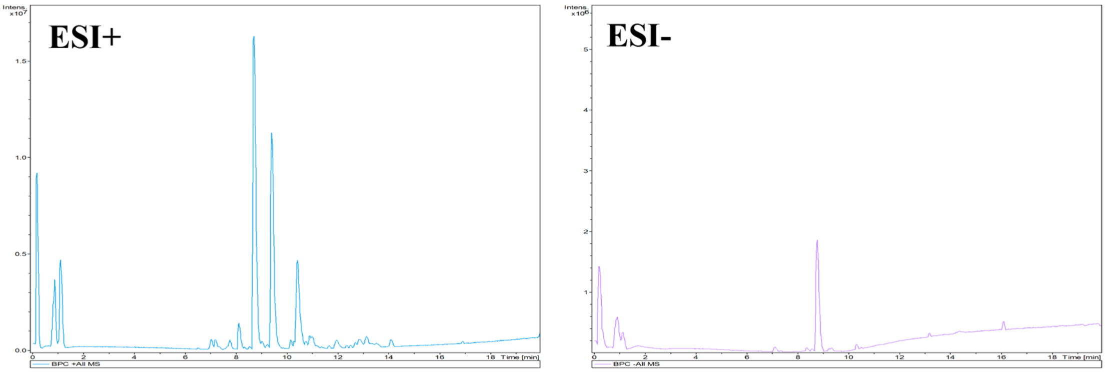

Figure S1. BPC profile of QC cell sample in positive (A) and negative (B) electrospray ionization mode.

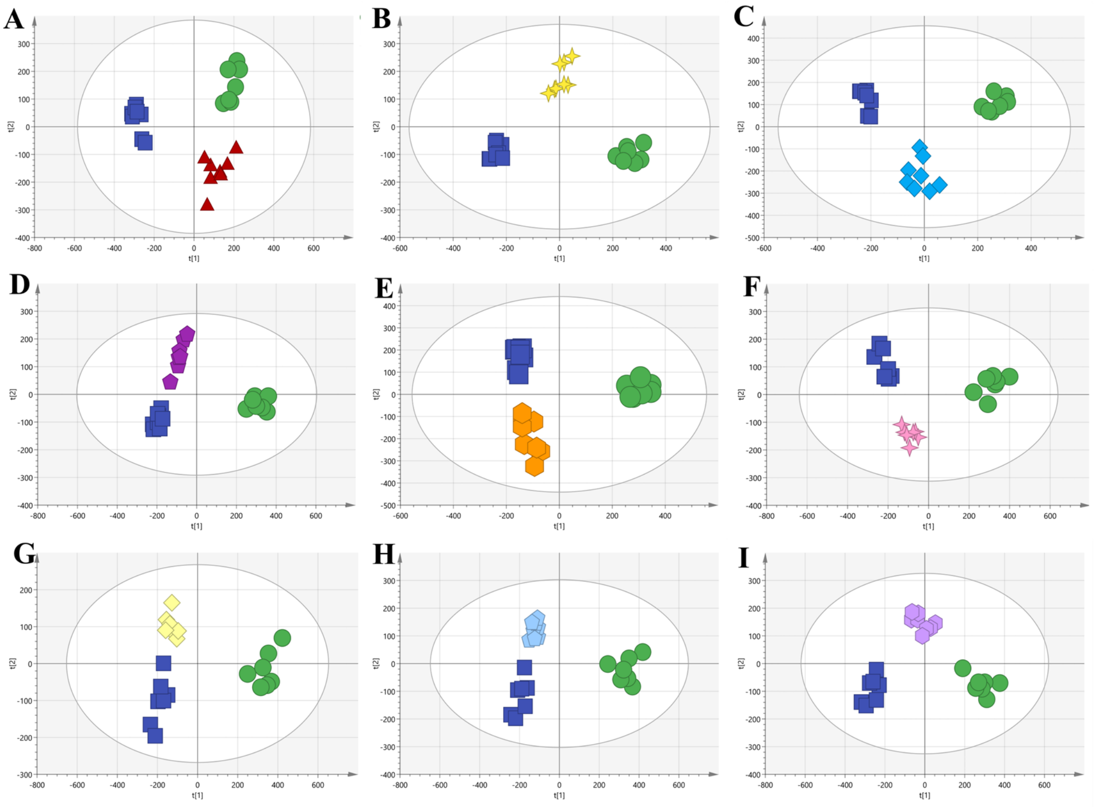

Figure S2. The PCA score plots obtained from NC, M and eight treatment groups (NC, GE, CAT, GPA, GEN, AU, AJU, RC, RD *VS* M).

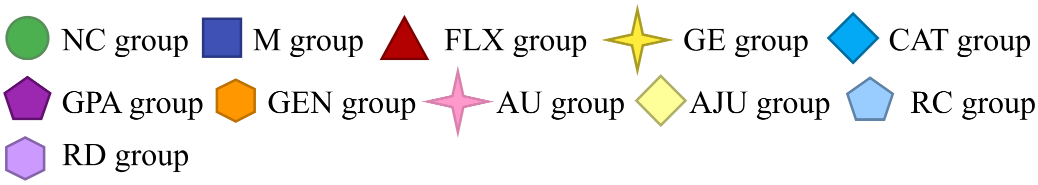

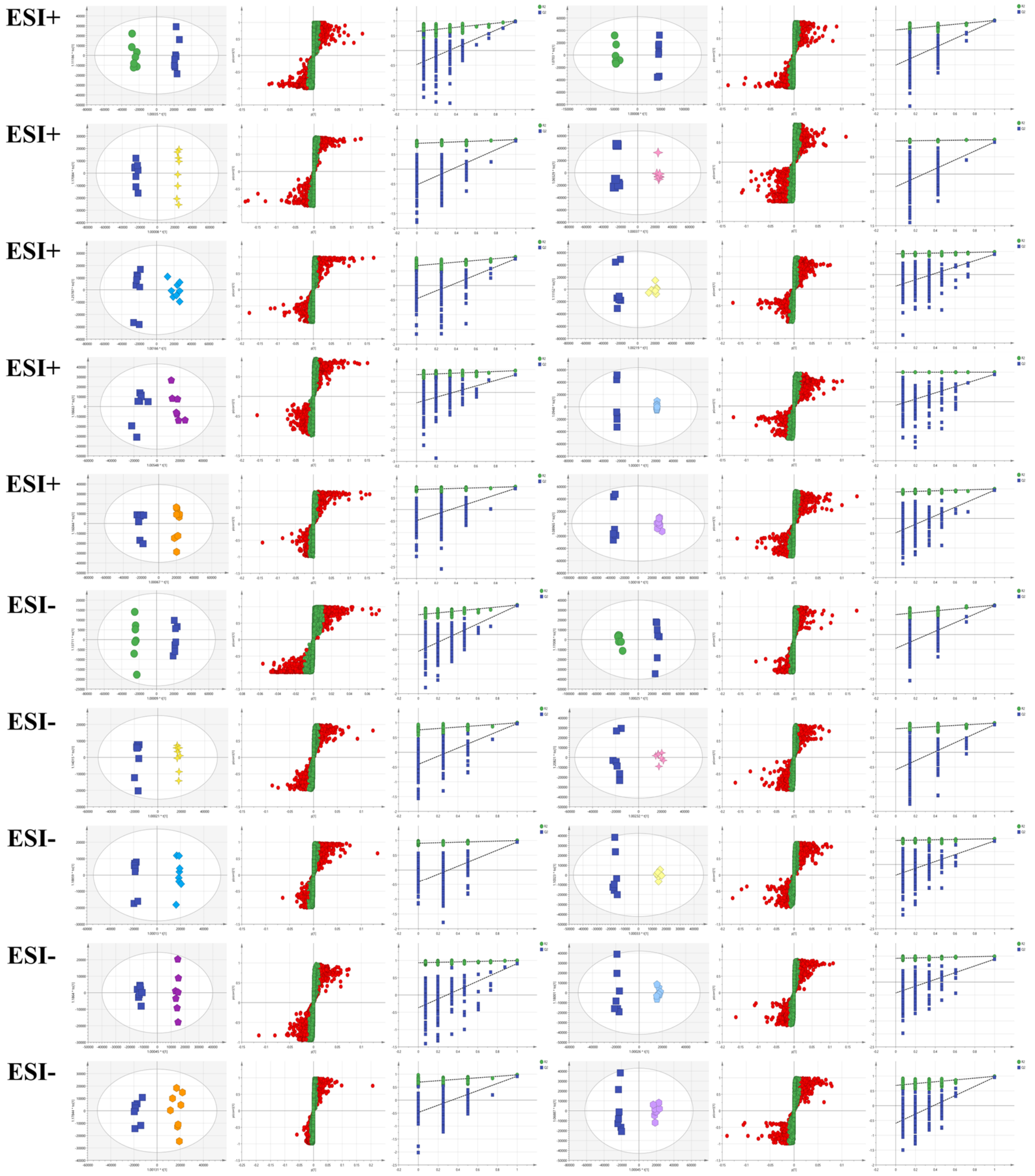

Figure S3. OPLS-DA scores, S-plots, validation plots derived from NC, M and eight treatment groups (NC, GE, CAT, GPA, GEN, AU, AJU, RC, RD *VS* M).

NC group M group FLX group GE group CAT group GPA group GEN group AU group AJU group RC group RD group

ESI+

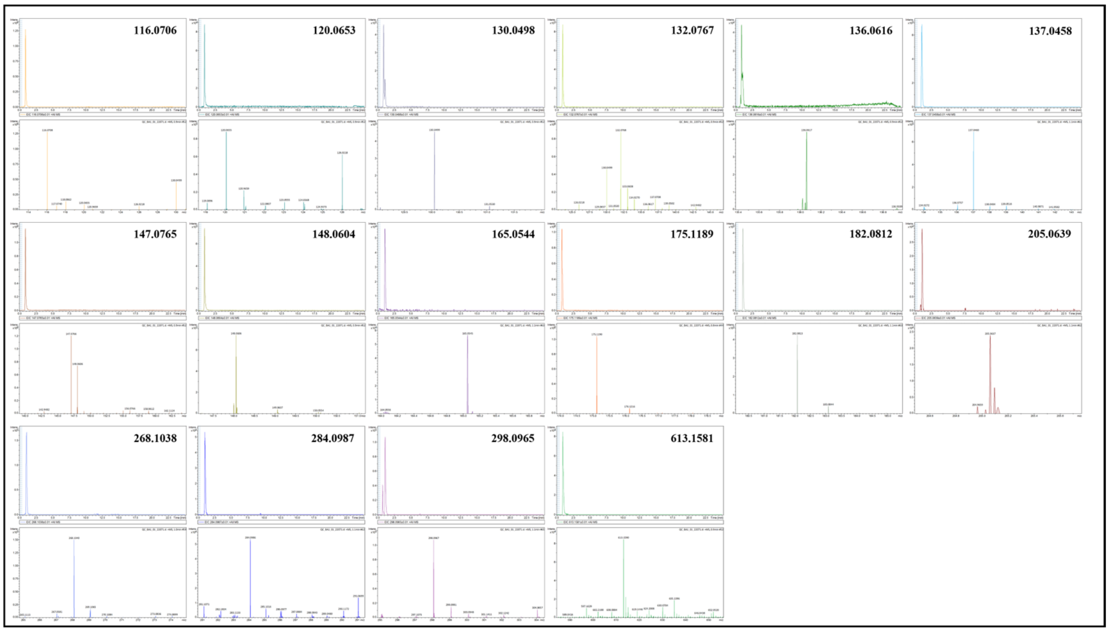

ESI-

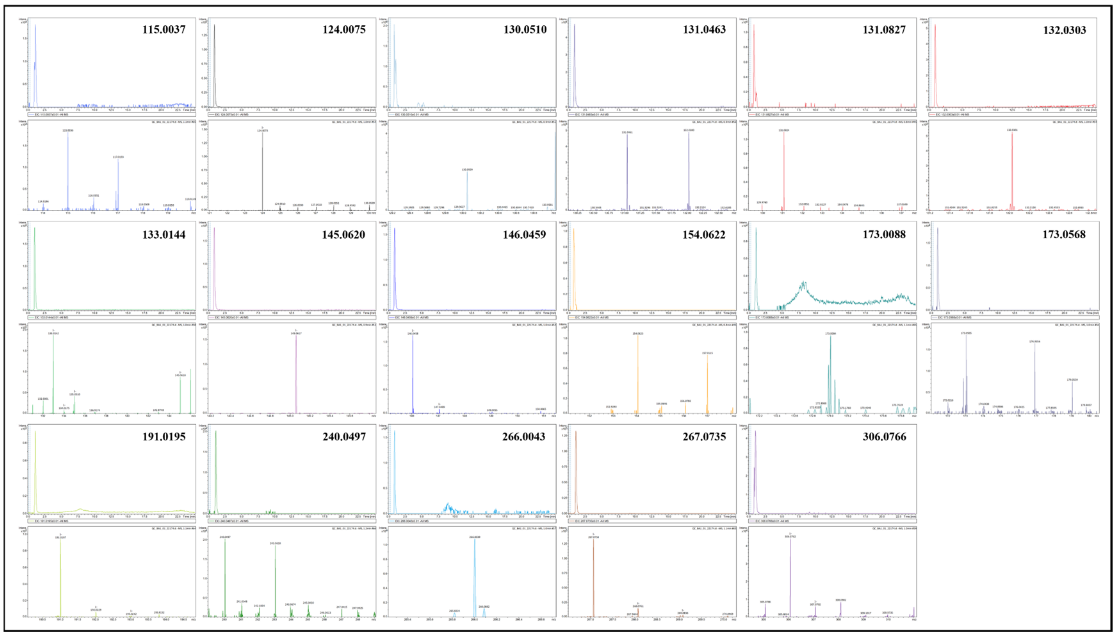

Figure S4. The EICs and MS spectra of pooled QCs for metabolites that co-eluted within the first minute in positive and negative ion modes respectively.

Table S1. RSDs of Retention Time and Peak Area of 38 metabolites in QC samples.

NO.	Adduct	*m/z*	Metabolites	RSDs of Rt (%)	RSDs of peak area (%)
1	M+H	116.0706	L-Proline	0.58	2.03
2	M+H	120.0653	L-Homoserine	0.63	1.97
3	M+ACN+H	130.0498	Pyruvic acid	0.56	1.84
4	M+H	132.0767	Creatine	0.47	2.53
5	M+H	136.0616	Adenine	0.55	4.62
6	M+H	137.0458	Hypoxanthine	0.97	4.11
7	M+H	147.0765	L-Glutamine	0.52	4.55
8	M+H	148.0604	L-Glutamic acid	0.50	3.95
9	M+H	165.0544	Phenylpyruvic acid	0.50	3.95
10	M+H	175.1189	L-Arginine	0.63	4.60
11	M+H	182.0812	L-Tyrosine	1.27	4.52
12	$M+H-H_2O$	205.0639	L-Cystathionine	0.84	4.99
13	M+H	268.1038	Adenosine	1.09	4.34
14	M+H	284.0987	Guanosine	1.46	4.06
15	M+H	298.0965	5'-Methylthioadenosine	0.98	3.31
16	M+H	302.3041	Sphinganine	0.04	1.22
17	M+H	347.2205	Corticosterone	0.06	1.02
18	M+ACN+Na	432.2359	Prostaglandin G2	0.04	4.73
19	M+H	613.1581	Oxidized glutathione	0.97	3.36
20	M-H	115.0037	Fumaric acid	0.25	3.43
21	M-H	124.0075	Taurine	0.62	1.99
22	M-H	130.051	4-Hydroxyproline	0.80	3.57
23	M-H	131.0463	L-Asparagine	0.71	3.58
24	M-H	131.0827	Ornithine	0.52	2.64
25	M-H	132.0303	L-Aspartic acid	0.70	4.10
26	$M-H_2O-H$	133.0144	Xanthine	0.49	2.58
27	M-H	145.062	D-Glutamine	0.54	2.36
28	M-H	146.0459	O-Acetylserine	0.68	2.64
29	$M-H_2O-H$	154.0622	2-Oxoarginine	1.09	3.77
30	$M-H_2O-H$	173.0088	Isocitric acid	0.41	2.36
31	M-H	173.0568	Formiminoglutamic acid	0.68	2.33
32	M-H	191.0195	Citric acid	0.44	2.34
33	M-H+HCOONa	240.0497	N-Acetyl-L-glutamate 5-semialdehyde	0.49	3.09
34	M-H+HCOONa	266.0043	O-Phosphohomoserine	0.67	3.62
35	M-H	267.0735	Inosine	0.59	2.45
36	M-H	303.2334	Arachidonic acid	0.04	1.36
37	M-H	306.0766	Glutathione	0.63	3.65
38	M-H	351.2174	Prostaglandin E2	0.06	3.11

Table S2. Summary of differential biomarkers.

NO.	Rt /min	*m/z*	Metabolites	NC *vs.* M	GE *vs.* M	CAT *vs.* M	GPA *vs.* M	GEN *vs.* M	AU *vs.* M	AJU *vs.* M	RC *vs.* M	RD *vs.* M	Formula	Adduct	Major ionic fragments
1	0.9	116.0706	L-Proline	√	√	√	√	√	√	√	√	√	$C_5H_9NO_2$	M+H	70.0477, 116.0713
2	0.9	120.0653	L-Homoserine		√	√	√	√					$C_4H_9NO_3$	M+H	101.5083
3	0.9	130.0498	Pyruvic acid	√	√	√	√	√	√	√	√	√	$C_3H_4O_3$	M+ACN+H	/
4	0.9	132.0767	Creatine	√	√	√	√	√	√	√	√	√	$C_4H_9N_3O_2$	M+H	111.0202
5	0.9	136.0616	Adenine	√	√	√	√	√	√	√	√	√	$C_5H_5N_5$	M+H	92.0237
6	1.1	137.0458	Hypoxanthine	√	√	√	√	√	√	√	√	√	$C_5H_4N_4O$	M+H	82.0727
7	0.9	147.0765	L-Glutamine	√	√	√	√	√	√	√	√	√	$C_5H_{10}N_2O_3$	M+H	146.9621
8	0.9	148.0604	L-Glutamic acid	√	√	√	√	√	√	√	√	√	$C_5H_9NO_4$	M+H	102.0542
9	1.1	165.0544	Phenylpyruvic acid	√	√	√	√	√					$C_9H_8O_3$	M+H	147.0458
10	0.9	175.1189	L-Arginine	√	√	√	√	√	√	√	√	√	$C_6H_{14}N_4O_2$	M+H	115.9628, 157.6914, 174.9356
11	1.1	182.0812	L-Tyrosine	√	√	√	√	√	√	√	√	√	$C_9H_{11}NO_3$	M+H	94.8877
12	1.1	205.0639	L-Cystathionine	√	√	√	√	√		√	√	√	$C_7H_{14}N_2O_4S$	M+H-H$_2$O	133.9545
13	1.1	268.1038	Adenosine	√		√	√	√		√	√	√	$C_{10}H_{13}N_5O_4$	M+H	136.0617
14	1.1	284.0987	Guanosine	√		√	√				√	√	$C_{10}H_{13}N_5O_5$	M+H	152.0395
15	1.3	298.0965	5'-Methylthioadenosine	√	√	√	√	√	√	√	√	√	$C_{11}H_{15}N_5O_3S$	M+H	93.9967, 119.0101
16	9.5	302.3041	Sphinganine	√	√	√	√	√	√	√	√	√	$C_{18}H_{39}NO_2$	M+H	241.2375, 254.2805
17	8.9	347.2205	Corticosterone	√	√	√	√	√	√	√	√	√	$C_{21}H_{30}O_4$	M+H	171.1136, 183.1130
18	10.5	432.2359	Prostaglandin G2	√	√	√	√	√					$C_{20}H_{32}O_6$	M+ACN+Na	279.1929, 321.2067
19	1.1	613.1581	Oxidized glutathione	√	√	√	√	√			√		$C_{20}H_{32}N_6O_{12}S_2$	M+H	484.1412, 612.8720
20	1.2	115.0037	Fumaric acid	√		√	√	√			√		$C_4H_4O_4$	M-H	114.9387
21	1.0	124.0075	Taurine	√	√	√	√	√	√	√	√	√	$C_2H_7NO_3S$	M-H	79.9538, 94.9715
22	1.0	130.051	4-Hydroxyproline	√	√	√	√	√	√	√	√	√	$C_5H_9NO_3$	M-H	112.0361, 128.0321
23	1.0	131.0463	L-Asparagine	√	√	√	√	√	√	√	√	√	$C_4H_8N_2O_3$	M-H	113.1976

24	0.8	131.0827	Ornithine	√	√	√	√	√	√	√	√	√	$C_5H_{12}N_2O_2$	M-H	130.8017
25	1.0	132.0303	L-Aspartic acid	√	√	√	√	√	√	√	√	√	$C_4H_7NO_4$	M-H	71.3029，88.1008
26	1.0	133.0144	Xanthine	√	√	√	√	√		√	√	√	$C_5H_4N_4O_2$	M-H_2O-H	108.0105
27	1.0	145.062	D-Glutamine	√	√	√	√	√	√	√	√	√	$C_5H_{10}N_2O_3$	M-H	84.0395
28	1.0	146.0459	O-Acetylserine	√	√	√	√	√	√	√	√	√	$C_5H_9NO_4$	M-H	65.9879，74.0336，114.1146
29	0.8	154.0622	2-Oxoarginine			√	√						$C_6H_{11}N_3O_3$	M-H_2O-H	128.0809
30	1.2	173.0088	Isocitric acid						√	√	√	√	$C_6H_8O_7$	M-H_2O-H	85.0265，111.0743
31	1.0	173.0568	Formiminoglutamic acid	√	√	√	√	√	√	√	√	√	$C_6H_{10}N_2O_4$	M-H	125.0284，146.0447
32	1.2	191.0195	Citric acid	√	√	√	√	√	√	√	√	√	$C_6H_8O_7$	M-H	110.9470，190.9737
33	1.2	240.0497	N-Acetyl-L-glutamate 5-semialdehyde			√	√	√					$C_7H_{11}NO_4$	M-H+HCOONa	130.0525，154.0518
34	1.2	266.0043	O-Phosphohomoserine	√	√	√	√	√		√	√	√	$C_4H_{10}NO_6P$	M-H+HCOONa	78.9503
35	1.2	267.0735	Inosine	√	√	√	√	√	√	√	√	√	$C_{10}H_{12}N_4O_5$	M-H	135.1022，229.3000
36	19.8	303.2334	Arachidonic acid	√	√	√	√	√	√	√	√	√	$C_{20}H_{32}O_2$	M-H	285.2112
37	1.0	306.0766	Glutathione	√	√	√	√	√	√	√	√		$C_{10}H_{17}N_3O_6S$	M-H	143.0974，179.5969
38	8.8	351.2174	Prostaglandin E2	√	√	√		√			√	√	$C_{20}H_{32}O_5$	M-H	271.1569，333.1529

Table S3. Fold Change report of differential biomarkers.

Metabolites	Fold Change (FC)									
	NC *vs* M	GE *vs* M	CAT *vs* M	GPA *vs*M	GEN *vs* M	NC *vs* M	AU *vs* M	AJU *vs* M	RC *vs* M	RD *vs*M
L-Proline	3.86	5.76	9.88	5.46	6.96	1.08	1.36	1.34	1.75	1.59
L-Homoserine	1.27	2.78	4.14	2.87	3.03	/	/	/	/	/
Pyruvic acid	4.04	5.56	8.49	5.31	5.91	1.07	1.36	1.26	1.48	1.25
Creatine	10.52	7.76	13.20	6.41	9.33	2.70	1.38	1.56	1.95	2.03
Adenine	1.64	1.70	2.64	1.87	2.84	1.57	1.13	1.58	1.43	2.08
Hypoxanthine	4.25	4.07	7.06	2.90	5.73	2.35	1.48	1.38	1.77	1.72
L-Glutamine	0.59	0.79	0.75	0.65	0.88	0.67	1.07	0.98	1.23	0.92
L-Glutamic acid	0.13	0.32	0.76	0.48	0.85	0.53	0.64	0.70	0.84	0.80
Phenylpyruvic acid	2.87	4.65	6.97	3.99	6.66	/	/	/	/	/
L-Arginine	4.10	4.06	5.05	3.02	5.08	1.23	1.05	1.05	1.16	1.20
L-Tyrosine	3.01	4.91	7.55	4.22	7.26	1.04	1.13	1.11	1.41	1.41
L-Cystathionine	3.03	3.05	3.25	1.13	1.62	1.68	1.68	1.69	2.01	2.63
Adenosine	3.91	1.07	4.18	5.38	3.00	3.23	1.19	1.94	2.53	2.56
Guanosine	5.81	1.96	6.12	5.21	4.57	2.67	1.08	1.43	1.88	1.80
5'-Methylthioadenosine	5.53	3.19	4.83	2.53	4.77	2.40	1.45	1.50	2.03	2.32
Sphinganine	0.80	0.65	0.68	0.76	0.86	0.86	0.65	0.66	0.83	0.88
Corticosterone	0.67	0.46	0.82	0.80	0.82	0.01	0.71	0.85	0.86	0.95
Prostaglandin G2	0.69	0.50	0.87	0.84	0.86	/	/	/	/	/
Oxidized glutathione	9.91	15.88	23.00	12.88	18.67	1.75	1.22	1.09	1.56	1.31
Fumaric acid	10.23	6.98	13.11	7.44	12.56	1.78	1.03	1.13	1.33	1.46
Taurine	5.08	6.03	12.33	7.05	8.76	1.13	1.37	1.52	1.91	1.63
4-Hydroxyproline	3.52	2.60	4.17	3.14	3.12	1.29	1.27	1.36	1.37	1.28
L-Asparagine	3.83	3.88	6.32	4.53	5.09	1.07	1.15	1.16	1.21	1.11

Ornithine	1.84	1.64	2.20	1.76	2.06	1.11	1.05	1.14	1.18	1.20
L-Aspartic acid	7.31	3.36	6.25	4.44	4.75	2.05	1.13	1.29	1.61	1.49
Xanthine	14.71	10.88	24.13	12.01	21.18	2.44	1.18	1.28	1.88	1.85
D-Glutamine	0.41	0.42	0.83	0.40	0.74	0.42	0.95	0.96	0.98	0.85
O-Acetylserine	6.70	5.28	9.39	6.01	7.13	1.25	0.98	1.11	1.36	1.23
2-Oxoarginine	1.86	2.23	4.09	2.70	3.10	/	/	/	/	/
Isocitric acid	/	/	/	/	/	2.96	1.08	1.06	1.38	1.73
Formiminoglutamic acid	2.18	3.80	6.04	4.20	4.93	1.24	3.77	2.92	5.77	1.94
Citric acid	17.84	6.18	10.56	6.76	8.88	5.06	1.10	1.13	1.62	2.06
N-Acetyl-L-glutamate 5-semialdehyde	1.51	2.15	2.83	2.30	3.09	/	/	/	/	/
O-Phosphohomoserine	3.26	5.56	8.68	5.39	6.06	1.76	1.08	1.22	1.64	1.49
Inosine	2.50	1.35	4.28	3.00	3.16	1.26	1.10	1.28	1.63	1.32
Arachidonic acid	0.24	0.93	1.32	0.92	1.30	0.26	0.82	0.79	0.83	0.68
Glutathione	18.07	7.05	16.07	6.76	6.58	2.11	0.29	0.20	0.25	1.14
Prostaglandin E2	8.97	4.28	5.82	2.55	7.26	4.68	0.78	1.03	1.35	1.64
